# Soldier phenotypic differences among 2 invasive and destructive *Coptotermes* species and their hybrids (Blattodea: Isoptera: Rhinotermitidae)

**DOI:** 10.1093/jisesa/iead095

**Published:** 2023-11-11

**Authors:** Jayshree S Patel, Thomas Chouvenc, Chia-Chien Wu, Hou-Feng Li, Nan-Yao Su

**Affiliations:** Department of Entomology and Nematology, Ft. Lauderdale Research and Education Center, Institute of Food and Agricultural Sciences, University of Florida, Ft. Lauderdale, FL, USA; Oncospark Inc., Jacksonville, FL, USA; Department of Entomology and Nematology, Ft. Lauderdale Research and Education Center, Institute of Food and Agricultural Sciences, University of Florida, Ft. Lauderdale, FL, USA; Department of Entomology, National Chung Hsing University, Taichung, Taiwan; Department of Entomology, National Chung Hsing University, Taichung, Taiwan; Department of Entomology and Nematology, Ft. Lauderdale Research and Education Center, Institute of Food and Agricultural Sciences, University of Florida, Ft. Lauderdale, FL, USA

**Keywords:** termite, morphology, F1 hybrid, identification, structural pest

## Abstract

With recent evidence of hybridization events in the field, the phenotypic traits of F1 hybrid colonies of 2 destructive subterranean termite species, *Coptotermes formosanus* Shiraki and *Coptotermes gestroi* (Wasmann) remain to be investigated. In this study, laboratory colonies of 2 conspecific pairings and 2 heterospecific pairings (hybrid F = ♀*C. formosanus* × ♂*C. gestroi*, hybrid G = ♀*C. gestroi *× ♂*C. formosanus*) were examined in Florida, USA, and in Taiwan. Colony nest architecture for both hybrids displayed disorganized carton materials compared to the defined trabecular carton of both parental species. Soldier head measurements were not a reliable approach for diagnostic purposes, as soldier morphometric traits widely overlapped across all mating combinations, except for hybrid F soldiers displaying abnormally long mandibles. Hybrid F soldiers’ mandibles also remained parallel when at rest. However, 4 qualitative morphological differences in soldiers were determined for diagnostic purposes. First, the fontanelle in both hybrids is horizontally ellipsoid whereas subcircular in *C. gestroi* and trianguliform in *C. formosanus*. Second, sclerotized striations along the postmental sulcus are present in *C. gestroi*, absent in *C. formosanus*, and intermediate in both hybrid soldier types. Third, each lateral margin of the fontanelle is flanked by 2 setae in *C. formosanus* and both hybrids, while a single seta resides on each side of the fontanelle in *C. gestroi*. Finally, *C. gestroi* and hybrid soldiers’ heads are characterized by a bulging vertex that is lacking in *C. formosanus*. Therefore, a combination of these 4 characteristics now allows for soldier identification of hybrid *Coptotermes*.

## Introduction

The movement of organisms around the world mediated by human activity has resulted in the establishment of invasive species, with a wide range of ecological and economic consequences (Simberloff et al. 2013). In addition, if 2 congeneric species evolved in allopatry, but now occur in sympatry as a result of anthropogenic activities, there is a potential for gene flow between them, in the absence of established mating barriers and postzygotic incompatibilities (Mooney and Cleland 2001, Crispo et al. 2011), with possibilities for the emergence of hybrid zones (Buggs 2007, San Jose et al. 2023). Depending on the hybrid organism and the degree of phenotypic similarities between the 2 parental species, there can be challenges in detecting such hybrid zones (Gay et al. 2008). Thus, the assessment of various phenotypic traits, including morphology, behavior, and physiological traits can provide important tools for identifying hybrid forms (Mendonça et al. 2014). However, the use of molecular methods is often necessary to confirm such hybridization events (Mallet 2005), especially when phenotypic traits are relatively conserved among congeneric species, and/or when intraspecies phenotypic variability is high.

Phenotypically different outcomes are possible for F1 hybrid individuals: (i) they can be morphologically intermediate between the 2 parental species, displaying characters of both parents or characters that represent a “middle-ground” between those of the parents, (ii) they can be more similar to one parent than the other, or (iii) they can display phenotypes that depart from either parental species (Rinderer et al. 1990, Santos et al. 2001, Trotta et al. 2010, Yee et al. 2013). Some hybridization events in insects have resulted in individuals displaying intermediate phenotypes such as in ants (Seifert 1991, 1999), butterflies (Mavárez et al. 2006), and bees (Rinderer et al. 1990), as hybrid forms can successfully establish in cases of high hybrid viability and fertility (Barton and Hewitt 1985, Mavárez et al. 2006). However, hybridization may in some cases disrupts developmental processes that would result in dysfunctional phenotypes with poor survival and reproductive capabilities (Lowe et al. 2015, Todesco et al. 2016). Finally, some transgressive phenotypes that depart from either parental trait can result in heterosis, which may increase fitness in terms of growth and fecundity of hybrid offspring (Andersen et al. 2006, Burke and Arnold 2001, Dittrich-Reed and Fitzpatrick 2013).

Hybridization cases have been studied in different groups of social insects like ants, bees, and termites (Scott Schneider et al. 2004, Feldhaar et al. 2008, Hartke and Rosengaus 2011, Chouvenc et al. 2015, Fournier and Aron 2021). However, detecting hybridization in social insects can sometimes be challenging owing to their inherent reproductive division of labor within a colony. Successful hybrid colony growth would rely on the phenotypic traits and vigor of the F1 offspring working caste (sterile individuals), but its reproductive success and fitness ultimately rely on the production of a viable and fertile F1 reproductive caste (alates). In addition, the developmental plasticity of social insects results in wide morphological differences among and within castes in a colony. As a result, hybrid phenotypes in social insects should be investigated at 2 distinct life stages of colonies, from sterile individuals of established colonies, and from reproductive individuals during dispersal flight events. In termites specifically, hybrid morphology was only examined in a single study where workers from laboratory-reared interspecies hybrids in the genus *Cubitermes* displayed intermediate morphologies in their enteric valves (Bouillon and Vincke 1973). In the few other studies that reported potential interspecific hybridization in a handful of termite species, the morphology of F1 individuals was never examined, as they all displayed poor viability in the laboratory (Aldrich and Kambhampati 2009, Hartke and Rosengaus 2011, Connétable et al. 2012, Wu et al. 2020). However, the hybridization between 2 subterranean termite species, *Coptotermes formosanus* Shiraki and *Coptotermes gestroi* (Wasmann) (Chouvenc et al. 2015) provides an exception, as laboratory-reared hybrid *Coptotermes* colonies established from field-collected alates displayed growth rate and population sizes similar to their parental species at 4 years of age (Patel et al. 2020).

Both *C. formosanus* and *C. gestroi* are subterranean termite species with strong invasive capabilities, and among the most destructive pests of wooden structures and urban tree canopies in tropical and subtropical regions around the world (Rust and Su 2012, Evans et al. 2013, Chiu et al. 2016, Chouvenc and Foley 2018). The native range for *C. formosanus* includes subtropical mainland China and Taiwan, while *C. gestroi* originates from tropical southeast Asia. Owing to human-mediated activities, their respective distributions have spread in the past 2 decades in metropolitan south Florida where both species now occur sympatrically (Chouvenc et al. 2016a). The potential for hybridization between these 2 species is possible owing to their simultaneous dispersal season (Chouvenc et al. 2017a) and interspecies courtship behavior during such events (Chouvenc et al. 2020, Mizumoto et al. 2021). They also occur in sympatry in 3 other locations: Hainan (China), southern Taiwan, and Oahu (Hawaii) and simultaneous dispersal flights have been reported in each area, with the exception of Hainan (Li et al. 2009, Chouvenc et al. 2017a, Tong et al. 2017).

Due to such favorable circumstances and the absence of prezygotic barriers, there is a possibility for hybridization between these 2 species in these regions. Such *Coptotermes* hybrid can pose a risk to urban areas in potential hybrid zones as they have equivalent damage potential to their parental species, a thermal tolerance that overlaps with both *C. formosanus* and *C. gestroi* (15–35°C) (Patel et al. 2019a) and are highly active in warm climates (Patel et al. 2019b). Recently, a survey of *Coptotermes* alates dispersing in Taiwan revealed the existence of field-established F1 and F2 hybrid colonies in the field, confirming the potential for a gene flow between the 2 species (Chen et al. 2023), and provided a morphological description of hybrid alates. Since both parental species have the potential to spread to non-native areas and hybridize, the establishment of hybrid populations may have long-term consequences on the pest status of *Coptotermes* (Su et al. 2017, Chouvenc and Li, 2023).

While genetic markers for diagnostic identification of the 2 parental species and their hybrids were developed (Chouvenc et al. 2017b) and applied for field detection of hybrid *Coptotermes* (Chen et al. 2023), a determination using morphological traits of the soldier caste could provide a reliable, rapid, and cost-effective alternative for hybrid *Coptotermes* identification for established colonies detected in the field. Morphological keys for termites usually only apply to the 2 castes with characteristic specific traits (alates and soldiers), as the worker caste morphology is often highly conserved beyond the generic level (Su et al. 1997). The investigation of hybrid alates in Taiwan revealed a phenotype that is intermediate to the 2 parental species, for their size, their overall cuticle color, and the shape of their antennal spots (Chen et al. 2023). In addition, the cuticular hydrocarbon profiles of hybrid workers are also intermediate between the 2 parental species (Chouvenc and Su 2017). However, morphological characters from the soldier caste have yet to be determined owing to the high variability in phenotypic morphologies observed in laboratory-reared colonies (Chouvenc et al. 2014). As 4-year-old colonies and older have since been obtained, we here seek to develop an identification protocol through the comparison of morphological characters in soldiers of such laboratory-reared hybrids and their parental species.

**Fig. 1.**
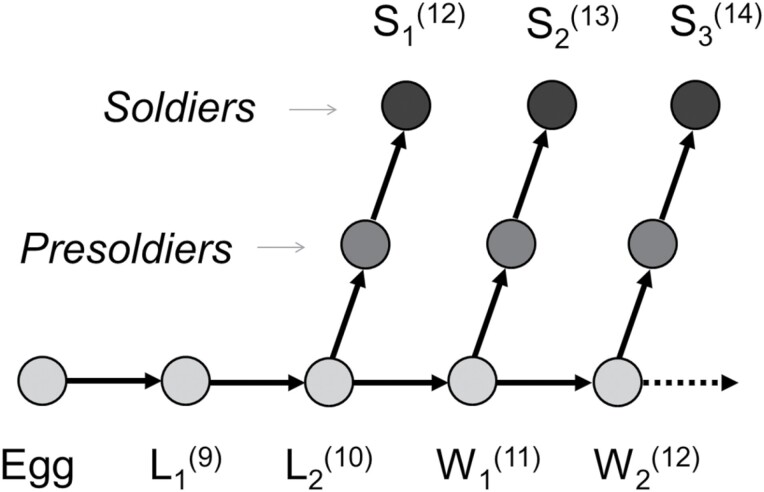
Differential developmental pathways for soldiers in *Coptotermes*, depending on the age of the colony. Each successive pathway includes an additional molt, resulting in relatively bigger individuals. Ln = instar larva, Wn = instar worker, and Sn = soldier pathway. Numbers in parenthesis indicate the numbers of antennomeres character of the instar/pathway.

From a morphometric approach, it can be unpractical to identify the soldier caste of *Coptotermes* at the species level using solely quantitative traits, owing to the inherent intracolonial and intraspecies variability, which has historically been a source of species identification confusion in *Coptotermes* (Chouvenc et al. 2016b). In addition, soldier developmental pathways are colony-age-dependent in *Coptotermes*, i.e., colonies at different ages produce soldiers through different pathways and a number of molts (Fig. 1). In incipient colonies, the second instar larvae (L_2_) molt into S_1_ (= nanitic) soldiers with 12 antennomeres and, as the colony ages, the soldier production shifts to the first instar worker (W_1_), followed by the second instar worker (W_2_), which produce S_2_ (13 antennomeres) and S_3_ (14 antennomeres), respectively (Chouvenc and Su 2014). Due to such complex developmental pathways, the soldier instars of different colonies at different ages vary greatly in size and in fluctuating asymmetry (Chouvenc et al. 2017c), making the identification process unreliable when measuring soldier head capsule traits.

However, the soldiers of the 2 parental species can be identified by qualitative traits: in *C. gestroi*, there is a bulging vertex behind the fontanelle in lateral view and 1 pair of setae beside the fontanelle, while in *C. formosanus*, this bulging vertex is absent and there are 2 pairs of setae beside the fontanelle (Scheffrahn and Su 2000). In addition, *C. gestroi* fontanelle is subcircular, while *C. formosanus* fontanelle is trianguliform (Chouvenc et al., 2014, Chen et al. 2020). However, diagnostic morphological traits remain to be determined in hybrid *Coptotermes* soldiers, for comparison with the 2 parental species and for rapid identification. The current study investigated 2 distinct morphological diagnostic approaches. First, a morphometric analysis using a set of measurements from 13 variables was performed to determine if hybrid soldiers display measurements that strongly deviate from either parental species. Second, qualitative traits were investigated in hybrid soldiers to key out hybrid soldiers from both parental species. In addition, having access to live colonies, this study was also able to investigate behavioral differences among mating combinations.

## Materials and Methods

### Termite Colony Rearing and Maintenance

*Coptotermes* soldiers investigated in this study were collected from laboratory-reared colonies of different ages established from 2014 to 2017 and the series of observations reported herein were made between 2017 and 2019. Laboratory colonies were established using field-collected alates during simultaneous dispersal flight events of *C. formosanus* and *C. gestroi* in Broward County, FL using light traps (Chouvenc et al. 2015) and in Taichung, Taiwan, using an insect net protocol (Chen 2022). Alates were temporarily placed in a container with moist corrugated cardboard overnight to favor self-dealation and transferred the next day to the laboratory for sex determination and species identification (Su et al. 1997). In the Florida laboratory, 1 male and 1 female dealate were paired into 4 mating combinations: ♀*C. gestroi *× ♂*C. gestroi* (= *C. gestroi*), ♀*C. formosanus* × ♂*C. formosanus* (= *C. formosanus*) and 2 heterospecific mating combinations: ♀*C. gestroi *× ♂*C. formosanus* (= hybrid G), ♀*C. formosanus* × ♂*C. gestroi* (= hybrid F), where the letter of the hybrid reflects the species of the queen. The pairs were then introduced in individual rearing units consisting of a transparent plastic cylindrical vial (8 cm high × 2.5 cm diameter, internal volume = 37 cm^3^) consisting of organic soil (Timberline topsoil, Oldcastle Lawn & Garden, Inc., Atlanta, GA), blocks of *Picea* sp., and 3% agar. As the colonies grew older, they were transferred in bigger size plastic containers of 1.5-L capacity (17.5 cm × 12.5 cm × 7 cm, Pioneer Plastics, Dixon, KY) at 2 years and colonies of 3 years and older were transferred in 13-L plastic containers (30.5 × 45.7 × 15.2 cm, Carlisle, Oklahoma City, OK). All the rearing units were maintained in a laboratory at 28 ± 2°C and regularly sprayed with water and supplied with additional wood as needed. Observations on the morphology of the carton nests of colonies from each mating combination were made. A modified rearing unit protocol (Chen 2022) was replicated in Taichung, Taiwan, to also obtain laboratory-reared colonies of all 4 mating combinations to support and complement observations of hybrid phenotypes in both locations. The measurements of soldiers’ head capsules were performed on termite material from Florida. Qualitative morphological and behavioral observations were made with termite material from both Florida and Taiwan.

### Processing of Soldiers

Soldiers were collected from 1-year-old to 4-year-old colonies of each mating combination and individually placed in 1.5-mL tubes with 95% ethanol. For each colony of origin (from 3 to 5 colonies of origin per mating combination, per each colony age), 4–5 soldiers were collected and the number of antennomeres was counted to confirm the developmental pathway of each individual soldier (Fig. 1). Soldiers missing terminal antennomeres on both antennae could not be placed in a given developmental pathway with certainty and were removed from the analysis. S_1_ soldiers with 12 antennomeres (= nanitic soldiers) were collected from 1-year-old colonies (*n* = 13–15 soldiers per mating combination for a total of 58 nanitic soldiers), S_2_ soldiers with 13 antennomeres were collected from 2-year-old colonies (*n* = 15–19 soldiers per mating combination for a total of 64 S_2_ soldiers) and S_3_ soldiers with 14 antennomeres were collected from 4-year-old colonies (*n* = 15–20 soldiers per mating combination for a total of 69 S_3_ soldiers).

**Fig. 2.**
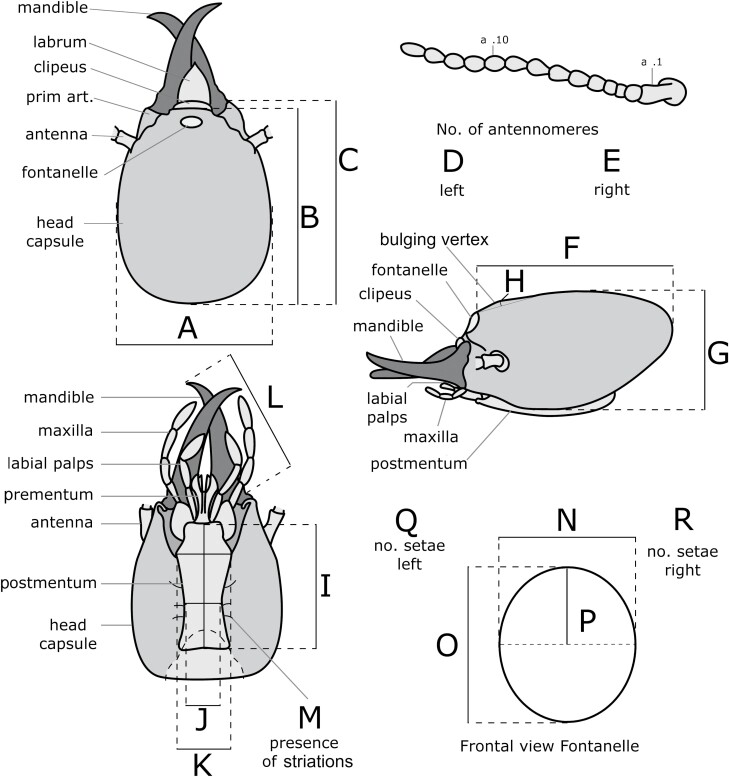
Different traits measured on the soldier head capsule. A) Head width, B) head length without clypeus, C) head length with clypeus, D) number of antennomeres on left, E) number of antennomeres on right, F) head length to fontanelle (lateral view), G) head height (lateral view), H) bulging vertex height (lateral view), I) postmentum length, J) postmentum width at center, K) postmentum width at base, L) mandible length, N) fontanelle width, O) fontanelle height, and P) fontanelle radius. Qualitative traits like M) presence of striations along the postmental sulcus, and Q and R) number of setae on left side and right side, respectively.

A series of measurements (quantitative approach) were made on all soldiers following a modified approach from Roonwald and Bose (1969) (Fig. 2), e.g.: head width (A), head length without clypeus (B), head length with clypeus (C), number of antennomeres (D and E), head length to fontanelle (F), head height (G), bulging vertex height (H), postmentum length (I), postmentum width at the center (J), postmentum width at the base (K), mandible length (L), fontanelle width (N), fontanelle height (O), and dorsal fontanelle radius (P). In addition, ratios among various variables were calculated (A/B, F/G, H/I, N/O, J/K, J/L, and C/L) to determine if some mating type of origin could result in distinct ratios that could be useful for diagnostic purposes. In a qualitative approach, traits like the presence of sclerotized striations along the postmental sulcus (M), the number of setae pairs beside the fontanelle (Q and R), and the overall geometrical shape of the fontanelle were determined. An Olympus camera DP70 connected to an OLYMPUS SZX12 microscope was used to take images and measurements were obtained using OLYMPUS Cell Sens Entry software. Visualization of the striation along the postmental sulcus was performed by using an oblique light so that the shadow from the striations accentuated their visibility. For figure production, specimens were photographed with a Keyence VHX-7000 microscope. Finally, behavioral observations of live soldier termites (only for S_3_ soldiers) were made, with 20 soldiers of each mating combination placed in individual 9-cm Petri dish, left for acclimation for 15 min before observations. Two traits were observed: the degree of closure of mandibles of soldiers when at rest, and their ability to bite and exude their frontal gland secretion through the fontanelle when poked at with a finger. While partially subjective and non-standardized, this protocol provided relevant observations on behavioral differences between the mating types, without a microscope, and when dealing with live individuals.

### Data Analyses

For each soldier’s developmental pathway, quantitative variables were obtained and compared using their potential overlapping range of measurements. Overlapping ranges were also checked for all the ratios of measurements described above. If the range of measurements or ratios overlapped among soldiers of different mating combinations, such trait or ratio was deemed not useful for species identification. The means of the morphometric measurements were also analyzed for each variable using one-way analysis of variance (ANOVA) with mating type as a factor. Significant differences were subjected to Tukey’s HSD test. To determine the patterns of variations between the parental species and hybrids and to visualize differences, a principal component analysis (PCA) was performed for each soldier type (S_1_, S_2_, and S_3_). The PCA was performed with 13 quantitative morphometric traits and biplot/scatter plots were generated. All the analyses were performed in R version 3.3.1 (R Team 2020) and package ade4 (Dray and Dufour 2007) was used for multivariate analyses. The “d” value in the PCA output figures represents the number of principal components retained in the transformed data. Additional figures and tables from the analysis were placed in the supplementary material (Supplementary Information S1) along with all raw measurements (Supplementary Information S2).

## Results

### Behavioral Observations

**Fig. 3.**
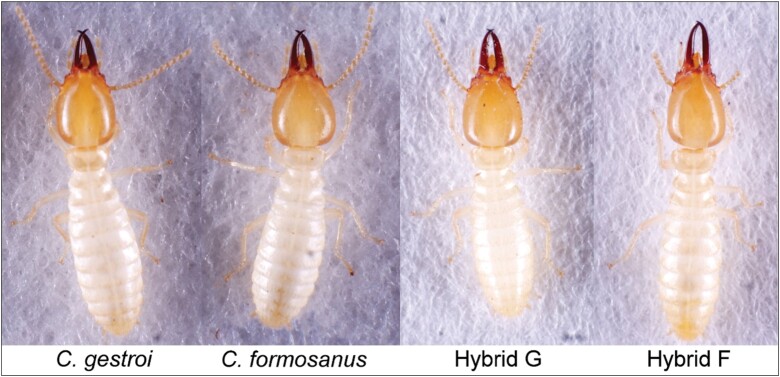
Dorsal view of mandibles position for soldiers at rest for all the 4 mating combinations with live specimens. Closed overlapping mandibles of *C. gestroi*, *C. formosanus*, and hybrid G (♀ *C. formosanus* × ♂*C. gestroi*). Note the relative parallel position of the mandibles at rest in hybrid F S_3_ soldiers (♀*C. gestroi *× ♂*C. formosanus*).

**Fig. 4.**
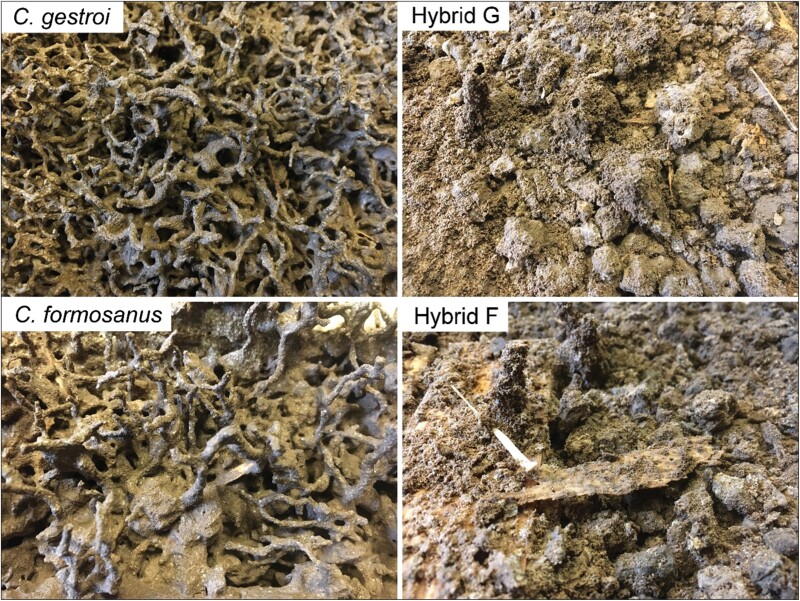
Variation in the external carton nest structures among the 4 mating combinations. Sponge-like structure of *C. gestroi* and *C. formosanus*. Scorched-earth texture with loose disorganized carton of hybrid G (♀*C. gestroi* × ♂*C. formosanus*) and hybrid F (♀ *C. formosanus* × ♂*C. gestroi*), respectively.

When placing live soldiers (S_3_) in a Petri dish and letting them acclimate for 15 min, a difference in posture of the mandibles was observed in soldiers of the hybrid F mating combination. First, soldiers of all mating combinations displayed the genus-characteristic tear drop-shape head capsules. However, even with the naked eye, the overall gestalt of the head capsule in hybrid F soldiers departed from either parental phenotype, appearing more elongated than soldiers from all other mating combinations (Fig. 3). While all soldiers displayed close, overlapping mandibles when at rest, hybrid F soldier displayed non-overlapping mandibles when at rest, resulting in mandibles being almost parallel (Fig. 3). From behavioral observations, when poked at with a finger, soldiers of both parental species were able to superficially penetrate the skin, latching onto the finger, and exuding copious amounts of defensive secretion from the frontal gland reservoir through the fontanelle (19 out of 20 trials for both species), which is a characteristic trait of *Coptotermes* soldiers. To some degree, hybrid G soldiers displayed variable difficulties in latching on the finger skin and exuded a minimal amount of defensive secretion, if any (7 out of 20 trials). In contrast, hybrid F soldiers were not capable of penetrating the skin, as their mandibles would slide on the surface when biting, propelling them backward when mandibles would close, in the manner of snapping mandibles. Hybrid F soldiers exuded their defensive secretion in few instances (only 1 out of 20 trials) when biting or being harassed, and when it did, the amount was minimal. These 2 behavioral differences in soldiers therefore allowed us to easily differentiate live hybrid F soldiers without a microscope, but were not useful traits to differentiate among the 2 parental species and hybrid G. Finally, a visual observation of the external nest texture of the carton material revealed a typical trabecular (sponge-like) structure observed in *C. gestroi* and *C. formosanus*. In contrast, carton material from the 2 hybrid types displayed superimposed layer patterns instead of mud tubes, which resulted in a “scorched earth” texture (Fig. 4), revealing that worker building behavior of both hybrid mating types departed from workers of either parental species.

### Soldiers Morphology

Because of the wide difference in soldier size among developmental pathways, the comparison among mating combination were independently performed for each of the S_1_, S_2_, and S_3_ types of soldiers. The analysis of morphological traits for S_1_ soldiers revealed that, despite the significant differences in the average of some variables (Supplementary Table S1), the extreme variability of measurements resulted in a wide overlapping of values among mating types for all quantitative variables and all tested ratios (Table 1, ratios overlap not shown). In addition, the strong fluctuating asymmetry observed in nanitic soldiers (Chouvenc et al. 2014) rendered most qualitative variables unusable, in particular, the number of setae varied greatly from 0 to 4 on either side of the fontanelle, which was itself highly variable in shape. One single qualitative trait stood out in S_1_ soldiers: the striations along the postmental sulcus (M) differed among mating combinations. In *C. formosanus* there was an absence of striations with a smooth appearance, while *C. gestroi* had 3–5 heavily sclerotized striations. Both combinations of S_1_ hybrid soldiers had a relatively higher number of striations (>6) than *C. gestroi* but were less sclerotized than in *C. gestroi*. This trait could not be used reliably to differentiate between the 2 S_1_ hybrid soldier types.

**Table 1.** Range of values (μm) for all morphometric traits of S_1_ soldiers of all 4 mating types: *C. gestroi*, *C. formosanus*, hybrid G (♀*C. formosanus* × ♂*C. gestroi*), and hybrid F (♀*C. gestroi* × ♂*C. formosanus*)

S_1_ soldier traits^a^	*C. gestroi*	*C. formosanus*	Hybrid G	Hybrid F
Number of replicates	*n* = 15	*n* = 15	*n* = 15	*n* = 13
A-Head width	876–1,048	945–1,197	886–1,063	922–1,150
B-Head length without clypeus	1,001–1,168	1,094–1,398	1,019–1,322	1,145–1,361
C-Head length with clypeus	1,040–1,209	1,133–1,446	1,048–1,400	1,184–1,464
F-Head length to fontanelle, LV	1,027–1,243	1,087–1,423	1,081–1,307	1,184–1,469
G-Head height, LV^b^	668–894	690–1,022	698–873	719–950
H-Bulging vertex height	31–68	27–45	36–61	40–69
I-Postmentum length	541–700	612–859	626–897	698–910
J-Postmentum width at center	185–344	205–239	187–241	201–229
K-Postmentum width at base	336–386	310–396	352–406	368–431
L-Mandible length	784–899	715–902	796–956	801–1,030
N-Fontanelle width	92–125	140–189	115–179	131–192
O-Fontanelle height	52–85	48–111	79–120	73–117
P-Fontanelle radius	23–48	26–67	38–73	34–69

^a^For S_1_ soldiers, all traits overlapped in measurements for all 4 mating types. See Supplementary Table S1 for further analysis.

^b^LV = Lateral view.

Similarly, for S_2_ soldiers_,_ some variables displayed significant differences in their averages (Supplementary Table S2) but all values for quantitative variables and ratios also widely overlapped among mating combinations for S_2_ (Table 2, ratio overlaps not shown). However, the overall fluctuating asymmetry was less pronounced than in S_1_, with a more distinct dorsally arched trianguliform fontanelle in *C. formosanus*, a subcircular fontanelle in *C. gestroi*, and a horizontally ellipsoid fontanelle in both hybrids. The number of setae still varied within mating types, but most samples displayed a “2 pairs” morphotype, regardless of mating combinations, including most *C. gestroi* S_2_ soldiers. Striations along the postmental sulcus reflected what was previously observed in S_1_, with an absence of striations in *C. formosanus*, few highly sclerotized striations in *C. gestroi*, and many slightly sclerotized striations in both hybrid types. In addition, the bulging vertex behind the fontanelle was absent in *C. formosanus*, while a weak bulging vertex was visible in *C. gestroi* and in both hybrid soldier types.

**Table 2.** Range of values (μm) for all traits of S_2_ soldiers of all 4 mating types: *C. gestroi*, *C. formosanus*, hybrid G (♀*C. formosanus* × ♂*C. gestroi*), and hybrid F (♀*C. gestroi *× ♂*C. formosanus*)

S_2_ soldier traits^a^	*C. gestroi*	*C. formosanus*	Hybrid G	Hybrid F
Number of replicates	*n* = 9	*n* = 15	*n* = 15	*n* = 15
A-Head width	932–1,150	1,056–1,196	1,021–1,225	945–1,179
B-Head length without clypeus	984–1,348	1,212–1,477	1,238–1,470	1,148–1,400
C-Head length with clypeus	1,007–1,400	1,251–1,544	1,276–1,531	1,179–1,467
F-Head length to fontanelle, LV	1,168–1,405	1,189–1,415	1,232–1,446	1,212–1,442
G-Head height, LV^b^	693–881	748–914	645–884	709–908
H-Bulging vertex height	30–68	27–49	45–57	37–63
I-Postmentum length	521–829	582–891	732–933	710–1,000
J-Postmentum width at center	207–270	208–262	196–250	189–220
K-Postmentum width at base	330–421	356–413	377–439	366–399
L-Mandible length	727–949	821–998	918–1,029	905–1,056
N-Fontanelle width	117–174	147–187	158–234	139–198
O-Fontanelle height	73–129	92–120	80–123	82–118
P-Fontanelle radius	38–69	49–68	42–66	42–68

^a^For S_2_ soldiers, all traits overlapped in measurements for all 4 mating types. See Supplementary Table S2 for further analysis.

^b^LV = Lateral view.

**Fig. 5.**
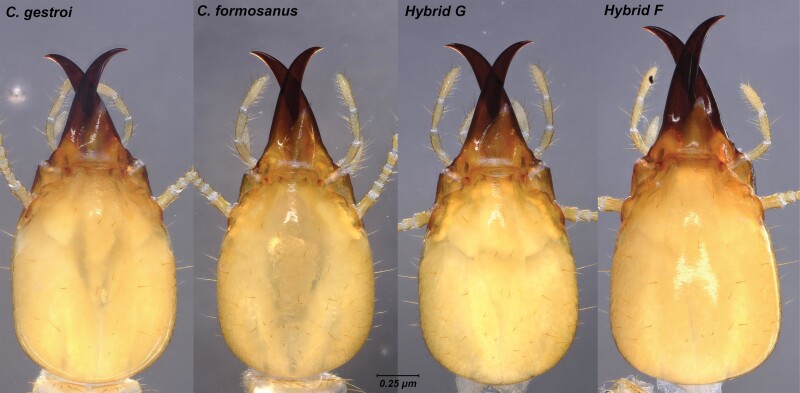
Dorsal view of S_3_ soldier head capsules from colonies of all 4 mating combinations. Note the relatively long mandibles in hybrid F soldiers and different head-shape gestalt when compared with the 3 other types of soldiers.

**Fig. 6.**
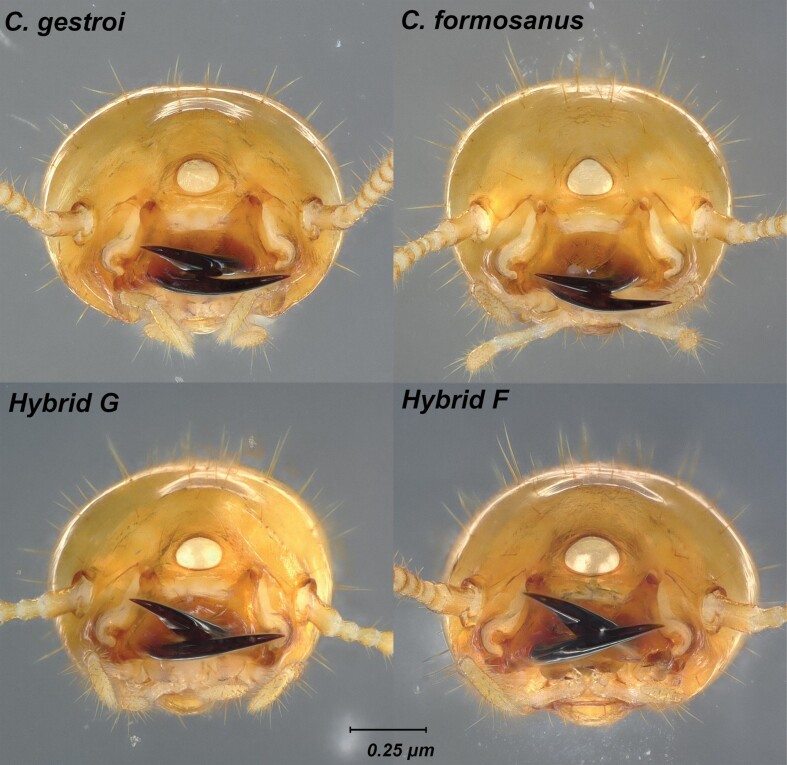
Frontal view of S_3_ soldier head capsules from colonies of all 4 mating combinations with a focus on the fontanelle shape. *Coptotermes gestroi* has a subcircular fontanelle. *Coptotermes formosanus* has a triaguliform fontanelle. Hybrid G (♀*C. gestroi *× ♂*C. formosanus*) and hybrid F (♀ *C. formosanus* × ♂*C. gestroi*) have a horizontally ellipsoid fontanelle.

**Fig. 7.**
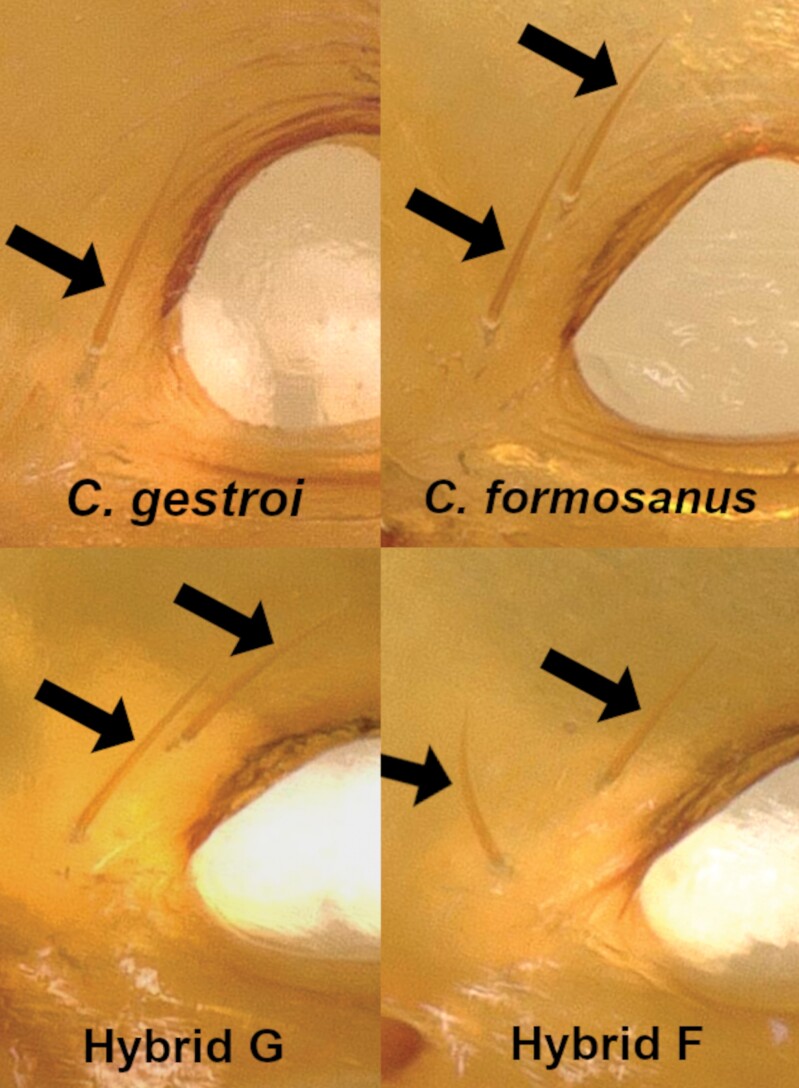
Close-up view of the seta(e) flanking the fontanelle from S_3_ soldier head capsules from colonies of all 4 mating combinations. Note a single pair of setae for *C. gestroi*, and 2 pairs for soldiers for all other 3 mating types.

**Fig. 8.**
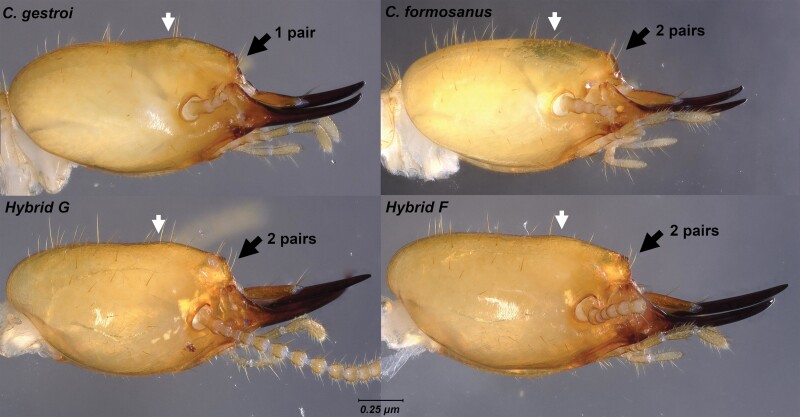
Lateral view of S_3_ soldier head capsules from colonies of all 4 mating combinations. The black arrows point to the number of pairs of setae beside the fontanelle, while the white arrows point to the bulging vertex behind the fontanelle, which is absent in *C. formosanus*. Note that the number of visible setae from lateral view is variable, as half of the setae can be obscured by the fontanelle. See Fig. 7 for visual confirmation of the typical number of setae expected for each mating combination.

**Fig. 9.**
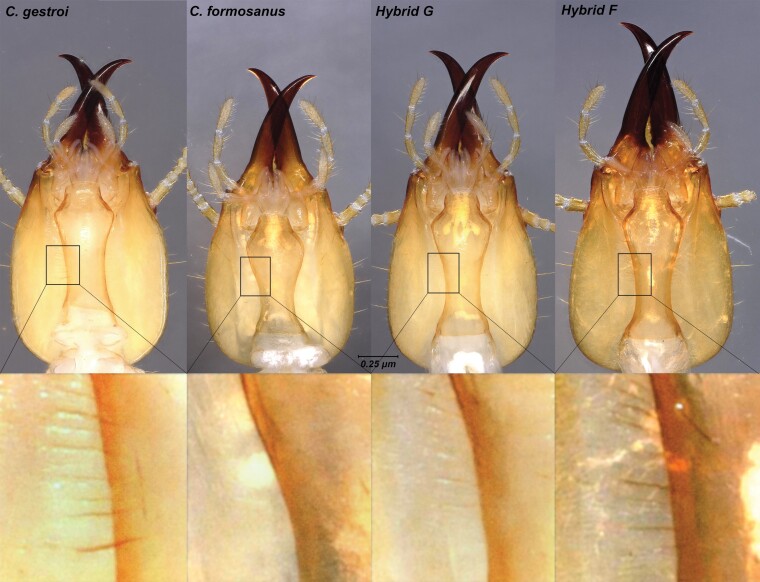
Ventral view of S_3_ soldier head capsules from colonies of all 4 mating combinations. Note the absence of striations along the postmental sulcus in soldiers of *C. formosanus*, heavy striations for *C. gestroi*, and variably faint striations for hybrid soldiers.

For S_3_ soldiers, again, some variables displayed a significant difference in their averages (Supplementary Table S3), but all variables and ratios also showed wide overlapping ranges (Table 3, ratio overlaps not shown), with 1 notable exception: the mandible length (L), was distinctly longer in hybrid F (942–1,112 μm) when compared to both parental species (853–929 μm in *C. formosanus* and 762–865 μm in *C. gestroi*). However, the range of values for the mandible length in hybrid G (892–1,017 μm) overlapped with all 3 other mating combinations (Fig. 5). From a qualitative observation, S_3_ soldiers from *C. gestroi* displayed a characteristic 1 pair of setae beside a subcircular fontanelle, while S_3_ soldiers from *C. formosanus* displayed a characteristic 2 pairs of setae beside dorsally arched trianguliform fontanelle. Both hybrid types S_3_ soldiers displayed 2 pairs of setae beside a defined horizontally ellipsoid fontanelle (Figs. 6 and 7). The bulging vertex behind the fontanelle was clearly noticeable in *C. gestroi* and in both hybrid types but absent in *C. formosanus* (Fig. 8). Finally, as observed for S_2_, the cuticle striations along the postmental sulcus of S_3_ soldiers remained highly sclerotized in *C. gestroi*, absent in *C. formosanus*, and present but variably sclerotized in both hybrid types (Fig. 9).

**Table 3.** Range of values (μm) for all traits of S_3_ soldiers of all 4 mating types: *C. gestroi*, *C. formosanus*, hybrid G (♀*C. formosanus* × ♂*C. gestroi*), and hybrid F (♀*C. gestroi *× ♂*C. formosanus*)

S_3_ soldier traits	*C. gestroi*	*C. formosanus*	Hybrid G	Hybrid F
Number of replicates	*n* = 16	*n* = 15	*n* = 18	*n* = 20
A-Head width	960–1,068	1,011–1,109	971–1,214	1,035–1,217
B-Head length without clypeus	1,171–1,323	1,161–1,356	1,199–1,524	1,328–1,503
C-Head length with clypeus	1,217–1,374	1,225–1,408	1,250–1,580	1,346–1,565
F-Head length to fontanelle, LV	1,114–1,261	1,124–1,397	1,202–1,446	1,284–1,446
G-Head height, LV^a^	652–763	706–835	698–884	763–851
H-Bulging vertex height	39–65	25–53	34–56	42–61
I-Postmentum length	670–847	702–939	782–940	801–1,081
J-Postmentum width at center	201–288	200–256	197–256	185–246
K-Postmentum width at base	348–392	350–411	378–456	362–433
L-Mandible length	762–865	853–929	892–1,017	**942**–**1,112**^b^
N-Fontanelle width	120–165	137–198	170–203	162–223
O-Fontanelle height	82–109	95–134	78–118	83–129
P-Fontanelle radius	42–62	51–90	43–68	41–69

^a^LV = Lateral view.

^b^Single variable with a range (in bold) that did not overlap with either parental species. See Supplementary Table S3 for further analysis.

**Fig. 10.**
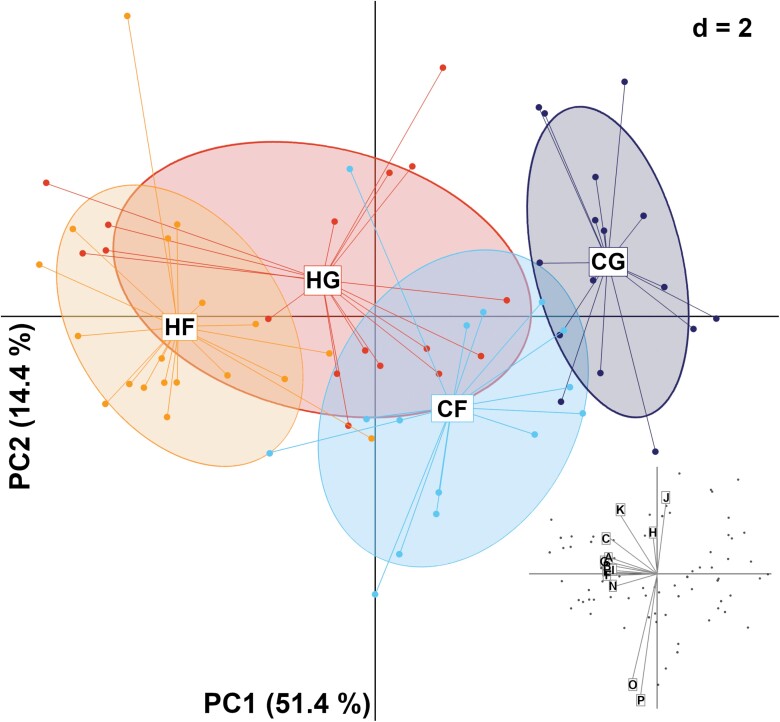
Principal component analysis (PCA) analysis of the 13 quantitative morphometric traits from S_3_ soldiers of the 4 mating combinations with HF (♀*C. formosanus* × ♂*C. gestroi*), HG (♀*C. gestroi* × ♂*C. formosanus*) (HG), *C. formosanus* (CF) and *C. gestroi* (CG), respectively. Scatter plots displaying the black dots that represent the data points for all mating types and vectors show the magnitude and direction of each trait contribution.

The PCA analysis of the morphological data confirmed a wide overlap of data points distribution with no clear distinction across soldiers from all 4 mating types, confirming that most intracolonial and intraspecies morphometrical variable overlapped across mating type, for S_1_ (Supplementary Fig. S1), S_2_ (Supplementary Fig. S2), and S_3_ (Fig. 10). For S_1_ and S_2_ soldiers, the first 3 components accounted for about 73% and 76% of the variation, respectively. For S_3_ soldiers, the first 3 components of the PCA analysis accounted for 75.1% of the observed variation in which the first component PC1 explained about half of the variation with 51.45% followed by PC2 = 14.43% and PC3 = 9.41%. The absence of clear segregation among the distribution of samples for each soldier type confirmed that the simple use of morphometric variables does not allow for directly determining the species identity of a soldier. On the contrary, qualitative traits allowed for such determination, especially for S_3_ soldiers.

## Discussion

The morphology of the soldier head capsule among *Coptotermes* species remains relatively conserved within all 4 species currently found in the neotropics, with some minor qualitative traits to tell them apart (Scheffrahn et al. 2015). As a result, morphometric parameters have not been used for diagnostic purposes (Scheffrahn and Su 2000, Chouvenc et al. 2016b, Wikantyoso et al. 2021) and historically, the species determination of *Coptotermes* soldier samples in Florida, Hawaii, and Taiwan was performed using the number of setae beside the fontanelle (1 pair for *C. gestroi*, 2 pairs for *C. formosanus*) and by the presence of a bulging vertex in *C. gestroi* (Scheffrahn and Su 2000). In addition, *C. formosanus* fontanelle is consistently trianguliform, while *C. gestroi* fontanelle is subcircular. (Chouvenc et al. 2014, Chen et al. 2020). In the current study, we were able to assess the potential use of morphologically qualitative and quantitative traits for species identity diagnostic among parental species and hybrid combinations, with a primary focus on S_3_ soldiers, as they would be the most likely soldier type to be found from field mature colonies of the 2 parental species (Chouvenc and Su 2014). A summary of all observed differences among mating types can be found in Table 4, which can now serve as a diagnostic tool for the identification of soldier samples from field colonies where both species distribution overlap (Su et al. 2017), and striations along the postmental sulcus was described as a new morphological trait to distinguish the 2 parental species.

**Table 4.** Summary of observed differences among all the 4 mating types: *C. gestroi*, *C. formosanus*, hybrid G (♀*C. formosanus* × ♂*C. gestroi*), and hybrid F (♀*C. gestroi* × ♂*C. formosanus*)

S_3_ soldiers’ characteristics^a^	*C. gestroi*	*C. formosanus*	Hybrid G	Hybrid F
Position of mandibles at rest	Overlapping	Overlapping	Overlapping	Parallel
Frontal gland secretion	Abundant	Abundant	Minimal	Minimal
Mandibles closing strength	Present	Present	Poor	Negligible
Mandible size range	Similar to *Cf*	Similar to *Cg*	Intermediate	Excessively long
Fontanelle shape	Circular	Almost triangular	Ellipsoid	Ellipsoid
Number of setae beside fontanelle	One pair	Two pairs	Two pairs	Two pairs
Bugle behind fontanelle	Present	Absent	Present	Present
Striations along postmental sulcus	Few, sclerotized	None, smooth	Many, variable	Many, variable
Carton nest structure	Trabecular	Trabecular	Disorganized	Disorganized

^a^Except for the last variable, which relates to a nest structure characteristic.

Behavioral observations also revealed that live soldiers from all mating combinations displayed closed, overlapping mandibles at rest except for hybrid F soldiers, which displayed a characteristic parallel position of the mandibles, with the tip the mandibles barely touching. In addition, it was possible to distinguish hybrid F soldiers from the 2 parental species by their moderate defensive abilities, potentially stemming from a weak strength while attempting to close their mandible, and also by their relative inability to exude defensive secretions. In comparison, hybrid G soldiers’ defensive abilities were intermediate between hybrid F soldiers and soldiers from either parental species which could both readily latch their mandibles and exude defensive secretion.

While these 2 behavioral traits (the open position of mandibles at rest for hybrid F, and weak defensive abilities), while anecdotical, could be useful characters to use from simple visual observations during field inspection, it cannot be used with dead, dried or alcohol-preserved samples, which is most often the way termites are provided for species identification requests. Similarly, while the difference in the texture of the carton nest allowed us to immediately tell if a colony belonged to a parental species (characteristic trabecular structure for both *C. gestroi* and *C. formosanus*), or a hybrid colony (poorly organized structure for both hybrid G and hybrid F), such trait may have minimal use in the field, because of the cryptic nature of *Coptotermes* nests. However, the differences in the nest structure observed between the hybrids and parental species imply that hybrid workers display a building behavior that results in poorly defined nest architecture, confirming some levels of altered behavioral functions in hybrid workers (Abbott et al. 2016, Lee et al. 2020). Such drastic differences in the expression of behavior in hybrid individuals compared to the fully expressed building behavior in both parental species could provide future opportunities to study the effect of the hybrid genetic makeup on altered social behaviors.

From a morphometric approach, the variability of soldier phenotypes resulted in extensive measurements overlapping across mating combinations, as confirmed by the PCA analysis. In addition, the intraspecies variability of measurements was also highly variable when investigating S_1_, S_2_, and S_3_ morphometric measurements. Therefore, this study confirms that a purely morphometric approach is not useful for diagnostic purposes in *Coptotermes*. with the notable exception of the length of the mandibles in hybrid F S_3_ soldiers, which were longer than soldiers from both parental species. Complex genetic interaction between 2 parental species can result in high variability and extreme phenotypes for the initial F1 generation but the effect can decrease in later generations of introgressive hybridization (Johansen-Morris and Latta 2006, Boel et al. 2019). The excessive length of hybrid F S_3_ soldier mandibles and their relatively poor ability to bite and produce defensive secretions may potentially imply a maladaptive transgressive phenotype for colonies to efficiently defend themselves and could therefore partially affect hybrid colonies’ fitness in a highly competitive or predative environment. However, such maladaptive traits may subdue in subsequent generations, and the existence of hybrid generations beyond F1 in the field provides opportunities to test such hypotheses in the future (Chen et al. 2023). In addition, as hybrid workers (Lee et al. 2020) and soldiers (current study) partially display transgressive phenotypes through altered developmental processes, hybrid colonies can provide biological material to further study mechanisms involved in caste determination and regulation in termites.

In conclusion, with the recent detection of hybrid alates in the field in Taiwan (Chen et al. 2023), it is expected to discover established hybrid colonies in the field in the near future, both in Taiwan and in Florida. While a purely morphometric approach was not reliable in identifying soldiers, this study determined 4 morphological characteristics useful for hybrid soldier identification purposes (Table 4). First, highly sclerotized striations along the postmental sulcus are present in *C. gestroi*, absent (smooth) in *C. formosanus*, and intermediate (variable striations) in hybrid soldiers. Second, the shape of the fontanelle is subcircular in *C. gestroi*, trianguliform in *C. formosanus*, and horizontally ellipsoid in both hybrids. Third, the number of setae beside the fontanelle is down to a single pair for *C. gestroi* but maintained at 2 pairs for *C. formosanus* and both hybrids. And fourth, *C. gestroi* and both hybrids display a bulging vertex behind the fontanelle, but it is absent in *C. formosanus*. In addition, with the excessive mandible length of hybrid F soldiers, all combined observations allow for the differentiation of soldiers from all 4 mating types.

## Supplementary Material

iead095_suppl_Supplementary_Tables_S1-S3_Figures_S1-S2

## Data Availability

All relevant data are included in the article and supporting information files: Supporting Information S1 (additional figures and tables) and S2 (raw morphometric data).
