# Supplementary material for: Soldier phenotypic differences among 2 invasive and destructive Coptotermes species and their hybrids (Blattodea: Isoptera: Rhinotermitidae)
Source: J Insect Sci. 2023 Nov 11;23(6):2. doi: 10.1093/jisesa/iead095 (PMC10640870; doi:10.1093/jisesa/iead095)

Supporting Information S1

Soldier phenotypic differences among two invasive and destructive *Coptotermes* species and their hybrids (Blattodea: Isoptera: Rhinotermitidae)

List of supplementary material in this file

Table S1. Statistical results from comparisons of S_1 soldiers

Table S2, Statistical results from comparisons of S_2 soldiers

Table S3, Statistical results from comparisons of S_3 soldiers

Figure S1. Principal component analysis of morphometric traits in S_1 soldiers

Figure S2. Principal component analysis of morphometric traits in S_2 soldiers

For Raw data, please see excel file, see Supplemental information S2

Table S1. Mean ± SD and results of analysis of variance for all the traits measured on the S_1 soldier type of the four mating types: *Coptotermes gestroi*, *Coptotermes formosanus,* Hybrid G (♀ *C. formosanus* × ♂*C. gestroi*) and Hybrid F (♀*C. gestroi* × ♂*C. formosanus*). All measurements in µm.

S_1 traits	*C. gestroi*	*C. formosanus*	Hybrid G	Hybrid F	F ratio	*df*	*P* value
A-Head width	942 ± 49b	1056 ± 71a	970 ± 57b	1073 ± 61a	16	3, 57	<.0001
B-Head length without clypeus	1085 ± 52c	1231 ± 112ab	1150 ± 94bc	1258 ± 70a	12	3, 57	<.0001
C-Head length with clypeus	1129 ± 58b	1298 ± 112a	1188± 110b	1324 ± 88a	13	3, 57	<.0001
F-Head length to fontanelle, LV	1151 ± 65c	1282 ± 94b	1217 ± 71bc	1361 ± 69a	20	3, 57	<.0001
G-Head height, LV	744 ± 61c	865 ± 89a	762 ± 53bc	832 ± 75c	10	3, 57	<.0001
H- Bulging vertex height	49 ± 11a	37 ± 5b	51 ± 7a	54 ± 8a	13	3, 57	<.0001
I- Postmentum length	612 ± 48c	738 ± 73b	737 ± 81b	828 ± 63a	24	3, 57	<.0001
J- Postmentum width at center	227 ± 36	220 ± 12	215 ± 14	219 ± 8	1	3, 57	0.47
K- Postmentum width at base	358 ± 16c	360 ± 23bc	376 ± 14ab	391 ± 15a	11	3, 57	<.0001
L- Mandible length	841 ± 35bc	829 ± 47c	882 ± 57b	940 ± 67a	13	3, 57	<.0001
N- Fontanelle width	110 ± 11b	160 ± 15a	146 ± 19a	162 ± 20a	31	3, 57	<.0001
O- Fontanelle height	72 ± 10b	91 ± 19a	98 ± 14a	94 ± 13a	10	3, 57	<.0001
P- Fontanelle radius	37 ± 7b	52 ± 14a	53 ± 10a	51 ± 10a	7	3, 57	<.0004

*Means followed by the same lowercase letter within a row are not significantly different (Tukey's HSD test, α = 0.05), four mating combination, 15 samples per mating combination.

Table S2. Mean ± SD and results of analysis of variance for all the traits measured on the S_2 soldier type of the four mating types: *Coptotermes gestroi*, *Coptotermes formosanus,* Hybrid G (♀ *C. formosanus* × ♂*C. gestroi*) and Hybrid F (♀*C. gestroi* × ♂*C. formosanus*). All measurements in µm.

S_2 traits	*C. gestroi*	*C. formosanus*	Hybrid G	Hybrid F	F ratio	*df*	*P* value
A-Head width	1043 ± 58c	1135 ± 46a	1095 ± 59ab	1073 ± 61bc	8	3, 63	<.0002
B-Head length without clypeus	1232 ± 84b	1341 ± 74a	1333 ± 72a	1301 ± 74ab	6	3, 63	<.0014
C-Head length with clypeus	1284± 91b	1384 ± 88a	1386 ± 81a	1354 ± 81ab	6	3, 63	<.0009
F-Head length to fontanelle, LV	1262 ± 66b	1317 ± 64ab	1316 ± 75ab	1341 ± 69a	4	3, 63	<.0089
G-Head height, LV	757 ± 48b	830 ± 39a	783 ± 64ab	803 ± 53ab	6	3, 63	<.0012
H- Bulging vertex height	50 ± 10a	40 ± 7b	49 ± 3a	51 ± 6a	8	3, 63	<.0001
I- Postmentum length	744 ± 85c	784 ± 89bc	834 ± 58ab	876 ± 84a	9	3, 63	<.0002
J- Postmentum width at center	237 ± 17a	233 ± 16ab	223 ± 17b	208 ± 8c	12	3, 63	<.0001
K- Postmentum width at base	378 ± 23b	392 ± 17ab	402 ± 22a	383 ± 9b	5	3, 63	0.0035
L- Mandible length	855 ± 58c	912 ± 44b	979 ± 34a	997 ± 47a	33	3, 63	<.0001
N- Fontanelle width	142 ± 15b	171 ± 13a	183 ± 21a	174 ± 15a	22	3, 63	<.0001
O- Fontanelle height	106 ± 12	110 ± 7	107 ± 11	101 ± 10	2	3, 63	0.0861
P- Fontanelle radius	57 ± 7	60 ± 6	57 ± 5	58 ± 7	1	3, 63	0.5075

*Means followed by the same lowercase letter within a row are not significantly different (Tukey's HSD test, α = 0.05), four mating combination, 15 samples per mating combination.

Table S3. Mean ± SD and results of analysis of variance for all the traits measured on the S_3 soldier type of the four mating types: *Coptotermes gestroi*, *Coptotermes formosanus*, Hybrid G (♀ *C. formosanus* × ♂*C. gestroi*) and Hybrid F (♀*C. gestroi* × ♂*C. formosanus*). All measurements in µm.

S_3 traits	*C. gestroi*	*C. formosanus*	Hybrid G	Hybrid F	F ratio	*df*	*P* value
A-Head width	1017 ± 37b	1056 ± 32b	1061 ± 74b	1127 ± 50a	14	3, 68	<.0001
B-Head length without clypeus	1243 ± 43b	1266 ± 53b	1363 ± 83a	1419 ± 57a	32	3, 68	<.0001
C-Head length with clypeus	1282 ± 43a	1318 ± 54a	1405± 97b	1460 ± 64b	25	3, 68	<.0001
F-Head length to fontanelle, LV	1201 ± 42c	1239 ± 73bc	1293 ± 80b	1368 ± 45a	25	3, 68	<.0001
G-Head height, LV	705 ± 29c	769 ± 38b	773 ± 53b	810 ± 29a	23	3, 68	<.0001
H- Bulging vertex height	52 ± 8a	41 ± 7b	48 ± 5a	50 ± 6a	9	3, 68	<.0001
I- Postmentum length	763 ± 47c	790 ± 63c	866 ± 45b	942 ± 71a	35	3, 68	<.0001
J- Postmentum width at center	247 ± 23a	229 ± 14b	227 ± 17b	223 ± 16b	6	3, 68	0.0011
K- Postmentum width at base	370 ± 15c	381 ± 19bc	409 ± 21a	398 ± 19ab	14	3, 68	<.0001
L- Mandible length	809 ± 23d	885 ± 24c	949 ± 35b	1028 ± 40a	149	3, 68	<.0001
N- Fontanelle width	144 ± 14c	167 ± 17b	185 ± 10a	187 ± 16a	31	3, 68	<.0001
O- Fontanelle height	100 ± 8b	113 ± 12a	105 ± 12ab	114 ± 11a	6	3, 68	0.0011
P- Fontanelle radius	53 ± 6b	64 ± 10a	58 ± 7ab	61 ± 6a	6	3, 68	0.001

*Means followed by the same lowercase letter within a row are not significantly different (Tukey's HSD test, α = 0.05), four mating combination, 15 samples per mating combination.

Figure S2. Principal component analysis (PCA) analysis of the thirteen quantitative morphometric traits from S_1 soldiers of the four mating types. The orange and coral ellipses represent the Hybrid F (♀ *C. formosanus* × ♂*C. gestroi*) (HF) and Hybrid G (♀*C. gestroi* × ♂*C. formosanus*) (HG) respectively and the sky blue and dark blue represent the parental species *C. formosanus* (CF) and *C. gestroi* (CG) respectively. Scatter plots display the black dots that represent the data points for all mating types and vectors show the magnitude and direction of each trait contribution.

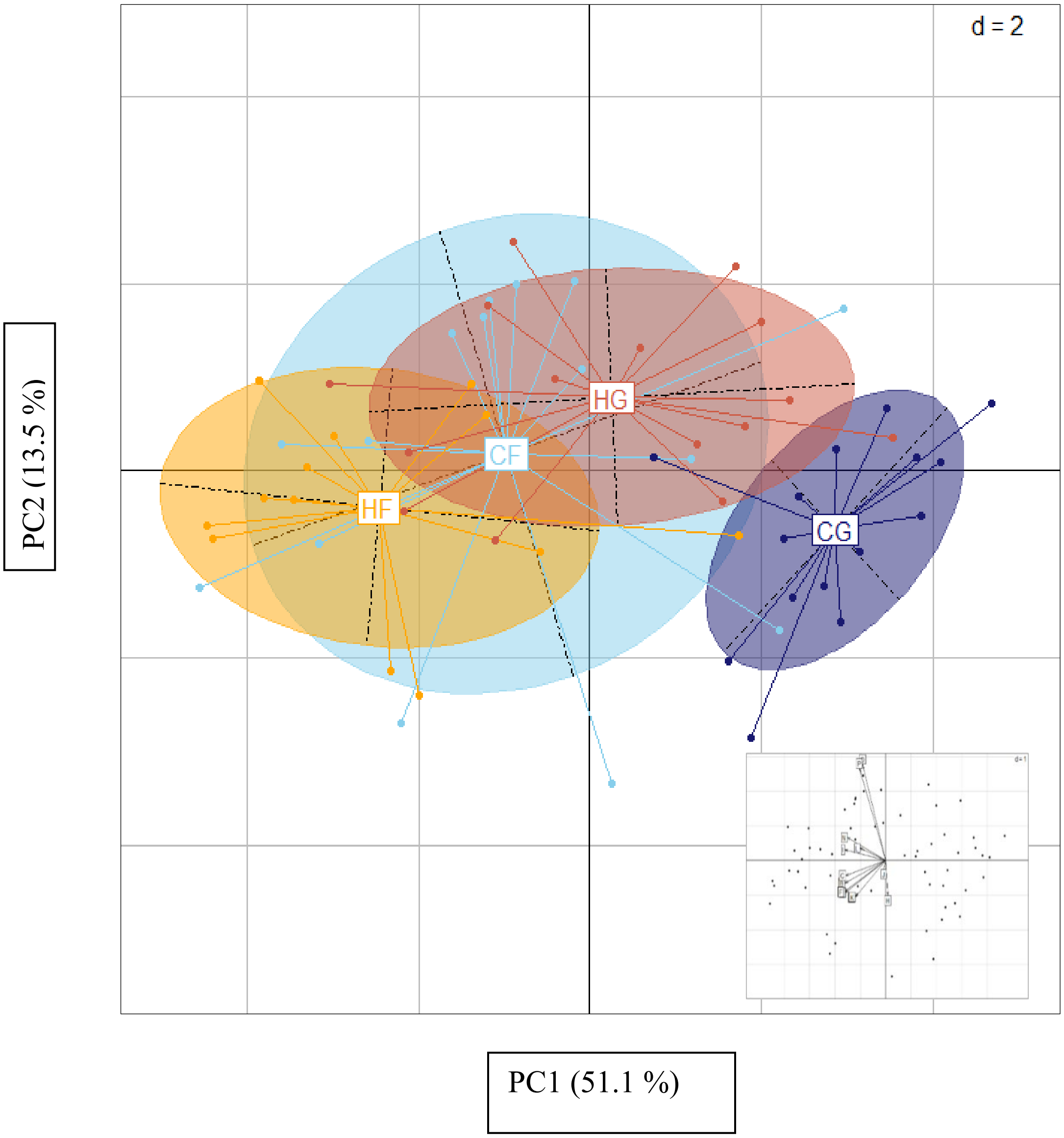

Figure S3. Principal component analysis (PCA) analysis of the thirteen quantitative morphometric traits from S_2 soldiers of the four mating types. The orange and coral ellipses represent the Hybrid F (HF) (♀ *C. formosanus* × ♂*C. gestroi*) and Hybrid G (♀*C. gestroi* × ♂*C. formosanus*) (HG) respectively and the sky blue and dark blue represent the parental species *C. formosanus* (CF) and *C. gestroi* (CG) respectively. Scatter plots display the black dots that represent the data points for all mating types and vectors show the magnitude and direction of each trait contribution.

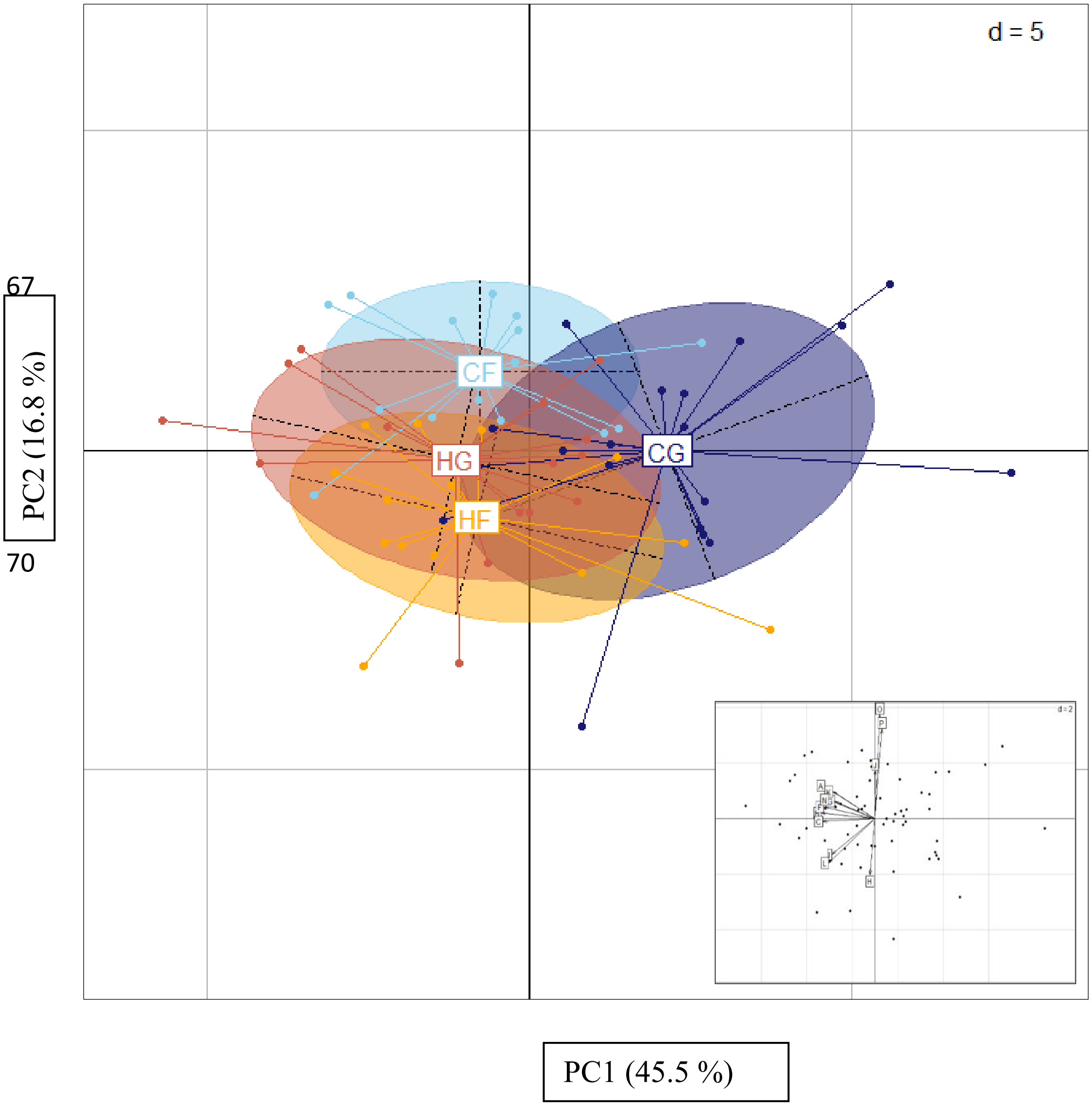

Supplement: iead095_suppl_Supplementary_Tables_S1-S3_Figures_S1-S2 [file iead095_suppl_supplementary_tables_s1-s3_figures_s1-s2.pdf]
